# Inactivated and Immunogenic SARS-CoV-2 for Safe Use in Immunoassays and as an Immunization Control for Non-Clinical Trials

**DOI:** 10.3390/v15071486

**Published:** 2023-06-30

**Authors:** Mariana Pierre de Barros Gomes, José Henrique Rezende Linhares, Tiago Pereira dos Santos, Renata Carvalho Pereira, Renata Tourinho Santos, Stephanie Almeida da Silva, Marta Cristina de Oliveira Souza, Juliana Fernandes Amorim da Silva, Gisela Freitas Trindade, Viviane Silva Gomes, Débora Ferreira Barreto-Vieira, Milena Mouta Verdan França Carvalho, Ana Paula Dinis Ano Bom, Noemi Rovaris Gardinali, Rodrigo Müller, Nathalia dos Santos Alves, Luma da Cruz Moura, Patrícia Cristina da Costa Neves, Gabriela Santos Esteves, Waleska Dias Schwarcz, Sotiris Missailidis, Ygara da Silva Mendes, Sheila Maria Barbosa de Lima

**Affiliations:** 1 Virological Technology Laboratory, Bio-Manguinhos/FIOCRUZ, Rio de Janeiro 21040-900, RJ, Brazil; mariana.gomes@bio.fiocruz.br (M.P.d.B.G.); jose.henrique@bio.fiocruz.br (J.H.R.L.); renata.carvalho@bio.fiocruz.br (R.C.P.); renata.tourinho@bio.fiocruz.br (R.T.S.); stephanie.silva@bio.fiocruz.br (S.A.d.S.); mcristina@bio.fiocruz.br (M.C.d.O.S.); juliana.silva@bio.fiocruz.br (J.F.A.d.S.); gisela.freitas@bio.fiocruz.br (G.F.T.); viviane.gomes@bio.fiocruz.br (V.S.G.); noemi.gardinali@bio.fiocruz.br (N.R.G.); nathalia.alves@bio.fiocruz.br (N.d.S.A.); luma.moura@bio.fiocruz.br (L.d.C.M.); waleska.dias@bio.fiocruz.br (W.D.S.); 2 Viral Morphology and Morphogenesis Laboratory, Oswaldo Cruz Institute/FIOCRUZ, Rio de Janeiro 21040-900, RJ, Brazil; barreto@ioc.fiocruz.br; 3 Immunological Technology Laboratory, Bio-Manguinhos/FIOCRUZ, Rio de Janeiro 21040-900, RJ, Brazil; milena.mouta@bio.fiocruz.br (M.M.V.F.C.); adinis@bio.fiocruz.br (A.P.D.A.B.); pcristina@bio.fiocruz.br (P.C.d.C.N.); 4 Pre-Clinical Trials Laboratory, Bio-Manguinhos/FIOCRUZ, Rio de Janeiro 21040-900, RJ, Brazil; rmuller@bio.fiocruz.br; 5 Recombinant Technology Laboratory, Bio-Manguinhos/FIOCRUZ, Rio de Janeiro 21040-900, RJ, Brazil; gabriela@bio.fiocruz.br; 6 Institute of Technology in Immunobiologicals, Bio-Manguinhos/FIOCRUZ, Rio de Janeiro 21040-900, RJ, Brazil; sotiris.missailidis@bio.fiocruz.br

**Keywords:** SARS-CoV-2 inactivation, beta-propiolactone, serial passages, TCID_50_, RT-qPCR, non-clinical trials, K18-hACE2 transgenic mice, PRNT_50_, immune response

## Abstract

Successful SARS-CoV-2 inactivation allows its safe use in Biosafety Level 2 facilities, and the use of the whole viral particle helps in the development of analytical methods and a more reliable immune response, contributing to the development and improvement of in vitro and in vivo assays. In order to obtain a functional product, we evaluated several inactivation protocols and observed that 0.03% beta-propiolactone for 24 h was the best condition tested, as it promoted SARS-CoV-2 inactivation above 99.99% and no cytopathic effect was visualized after five serial passages. Moreover, RT-qPCR and transmission electron microscopy revealed that RNA quantification and viral structure integrity were preserved. The antigenicity of inactivated SARS-CoV-2 was confirmed by ELISA using different Spike-neutralizing monoclonal antibodies. K18-hACE2 mice immunized with inactivated SARS-CoV-2, formulated in AddaS0_3_^TM^, presented high neutralizing antibody titers, no significant weight loss, and longer survival than controls from a lethal challenge, despite RNA detection in the oropharyngeal swab, lung, and brain. This work emphasizes the importance of using different techniques to confirm viral inactivation and avoid potentially disastrous contamination. We believe that an efficiently inactivated product can be used in several applications, including the development and improvement of molecular diagnostic kits, as an antigen for antibody production as well as a control for non-clinical trials.

## 1. Introduction

COVID-19, caused by the severe acute respiratory syndrome coronavirus 2 (SARS-CoV-2), was responsible for over 6,945,000 deaths worldwide until June 2023 [1]. The World Health Organization (WHO) declared COVID-19 as a public health emergency in early 2020 and, in parallel with the Center for Disease Control and Prevention (CDC), issued Laboratory Safety Guidelines for SARS-CoV-2 handling [2,3].

On 5 May 2023, due to the decline in the number of intensive care unit hospitalizations and deaths related to COVID-19, the World Health Organization declared the end of a Public Health Emergency of International Importance (PEMI) [4]. However, even with the availability of 30 vaccines in use [1] and 70.3% of the world’s population immunized with at least 1 dose [5], the continuous appearance of new variants raises a health risk that should not be overlooked. As a result, it is still recommended that all material with a high viral load (viral isolation and propagation, neutralization assays, or large volumes of infected material) be handled only in Biosafety Level 3 (BSL-3) facilities and by trained personnel [2,3]. In this context, safety is one of the main limiting factors in SARS-CoV-2 studies due to the high risk of transmission and exposure of healthcare professionals and scientists. Considering this scenario, successful viral inactivation is a good strategy as it allows the viral samples to be transferred from a BSL-3 facility to a Biosafety Level 2 (BSL-2) facility [6], enabling safe use.

The whole inactivated virus can be used as an antigen in immunological assays, vaccine preparations, and viral composition analysis [7,8,9]. There are different viral inactivation methods described, either by physical agents (e.g., pH and heat) or chemical agents (e.g., chaotropic salts, detergents, and aldehyde-based solutions) [8,10]. Heat inactivation is compatible with serum sample inactivation protocols for serological assays and it has long been used to inactivate viruses, including SARS-CoV-2, but it does not always preserve the viral particle, thus preventing the formation of immunocomplexes [11,12,13,14]. Approaches employing chemical agents, such as ascorbic acid combined with Cu(SO_4_)_2_, have been successfully used to inactivate the rabies virus, herpesvirus, paramyxovirus, and vaccinia virus [15,16,17], which does not affect the antigenicity of the viral particle [17] and can be adopted as an inactivator by low-cost and simple handling. Furthermore, glutaraldehyde is commonly used to disinfect hospital supplies that cannot be treated with heat or high pressure. It has also been used to inactivate the hepatitis A virus, poliovirus, SARS-CoV, and SARS-CoV-2 [6,18,19,20], and its use as a viral inactivator has also been demonstrated in preparations of hemoglobin-based oxygen carriers [21]. Beta-propiolactone has emerged as a potent inactivating agent widely used in the manufacture of inactivated vaccines, including for SARS-CoV-2-approved vaccines [22,23,24], since it can preserve the structure and antigenicity of viral particles. Advantages and disadvantages of each agent, physical or chemical, should be carefully considered according to the intended use of the inactivated virus.

For molecular diagnosis purposes, the virus can be used as input in serum panels for RT-qPCR validation assays or as positive extraction controls. In these cases, it is interesting to maintain the particle integrity, avoiding genome exposure to nucleases present in the medium, even if denaturation of viral proteins would have no impact on the results. Conversely, if the goal is to use the whole particle for vaccine production or in techniques involving the formation of immunocomplexes, such as ELISA, the maintenance of the three-dimensional structure and integrity of the viral particle are critical [25]. Therefore, selecting the best inactivation strategy requires a detailed understanding of each study, as no single agent or method works for all applications, and the activity of each inactivation agent should ideally be tested for each matrix that will be used in the study.

In this work, several inactivation protocols were tested to determine the best methodology for application in different areas of research and diagnosis, including heat and chemical inactivation with beta-propiolactone (βPL), glutaraldehyde (GLU), and ascorbic acid (ASC), all used in SARS-CoV-2 culture supernatant. To ensure virus inactivation, all treated samples were evaluated by TCID_50_ and RT-qPCR after successive cell culture passages. Among the agents tested, we found that βPL (0.03% for 24 h), a molecule widely used in vaccine development, was the best SARS-CoV-2-inactivation strategy, as it maintained the integrity of the virus particles and was able to elicit an efficient immune response in the murine model.

## 2. Materials and Methods

All reagents used were of analytical grade. Cultures of SARS-CoV-2, as well as initial processing of oropharyngeal swab and organ samples from the non-clinical trial, were handled in a BSL-3 laboratory, and the procedures from the challenge of animals with infectious SARS-CoV-2 (Wuhan strain) were conducted in an Animal Biosafety Level 3 (ABSL-3) facility, in accordance with approved international laboratory biosafety guidelines [2,3].

### 2.1. Cell Culture

Vero CCL-81 (ATCC, Manassas, VA, USA) and Vero E6 (CRL-1586, ATCC, Manassas, VA, USA) cells were maintained in medium 199 (Gibco, Billings, MA, USA), supplemented with 5% and 10% fetal bovine serum (FBS; Gibco, Billings, MA, USA), respectively, and 40 µg/mL of gentamicin sulfate (Santisa, Bauru, SP, Brazil). For Vero E6, the concentration of L-glutamine (Sigma-Aldrich, Saint Luis, MO, USA) was adjusted to 2 mM. Both cell lines were kept under 5% CO_2_ at 37 °C.

### 2.2. Virus Production

SARS-CoV-2 strains, Wuhan (GISAID EPI_ISL_414045), Alpha (B.1.1.7 lineage; GISAID EPI_ISL_1402430), Gamma (P.1 lineage; GISAID EPI_ISL_1402431), and Zeta (P.2 lineage; GISAID EPI_ISL_792642), were produced at MOI 0.01 in pre-formed stationary cultures of Vero E6 cells (density 70,000 cells/cm^2^), prepared 24 h prior to infection, to obtain 4 virus working banks in the presence (Wuhan and variant strains) and 1 in the absence of FBS (Wuhan strain). After the adsorption step at 37 °C for 1 h, the inoculum was completely removed, and medium 199, supplemented with 2% FBS and 2 mM of L-glutamine, or OptiPRO SFM (Gibco, Billings, MA, USA), supplemented with 4 mM of L-glutamine, was added. Incubation for viral production occurred in a CO_2_ incubator at 37 °C. All viral stocks were harvested 2 days post-infection (dpi). Viral suspension was clarified using sterilizing filtration with a 0.22 µm pore (Merck, Darmstadt, HE, Germany), and D-Sorbitol (Sigma-Aldrich, Saint Luis, MO, USA) at an 8% *m*/*v* final concentration was added to ensure virus stability at low temperatures. The viral stocks were kept at −80 °C.

### 2.3. SARS-CoV-2 Inactivation

Several chemical and physical inactivation protocols were evaluated (Table 1). The heat inactivation (at 56 °C, 65 °C, or 75 °C for 30 min) was conducted in conical tubes (15 mL) or microtubes (1.5 mL) containing 5 or 0.3 mL for each condition, respectively. The water bath was preheated until the desired temperature stabilized, which was confirmed by an external mercury thermometer. At each time point, the tubes were removed from the water bath and placed into an ice bath to match the temperature of the control samples. The ascorbic acid (Isofar, Duque de Caxias, RJ, Brazil) treatment was performed using two different concentrations (0.5 or 1.0 mg/mL) for 0 to 96 h at 4 °C. ASC inactivation was conducted in the presence of 5 µg/mL of Cu(SO_4_)_2_ (Merck, Darmstadt, HE, Germany). Glutaraldehyde (Merck, Darmstadt, HE, Germany) inactivation was performed using a concentration of 0.004% for 0 to 48 h at 4 °C. SARS-CoV-2 was also submitted to 0.03% β-propiolactone (Natalex, Warsaw, Masovian Province, Poland) for 0 to 48 h at 4 °C. Immediately after each time point, samples were incubated at 37 °C for 2 h to ensure complete hydrolysis of the βPL and minimize toxicity. For each condition, samples in the absence or presence of the virus were subjected to the same parameters and used as controls for each assay. Finally, all samples were kept at −80 °C until the infectivity was assessed (see Section 2.4).

In order to obtain a multifunctional product for safe use in a BSL-2 environment, the best inactivation protocol was determined by the following selection criteria: (1) inactivation > 99.9% (Log_10_ TCID_50_/mL), (2) maintenance of RNA quantification (Log_10_ RNA copies/mL) to enable its use in molecular biology tests, (3) a shorter incubation time, (4) absence of the cytopathic effect (CPE), (5) decreasing of viral RNA copies over serial passages in cell culture to confirm the inactivation process (see Section 2.7), and (6) viral structure integrity. All protocols were conducted in independent duplicates using a SARS-CoV-2 (Wuhan strain) working bank produced with FBS. Then, the best protocol was used to inactivate the other viral batches.

### 2.4. Virus Quantification by Median Tissue Culture Infectious Dose (TCID_50_)

The infectivity of viral stock and inactivation test samples was determined by the TCID_50_ assay based on the Kärber method [26] and quantified as previously reported [27]. Ten-fold serial dilutions of the virus at non-cytotoxic concentrations (10^0^ to 10^−6^ for TEMP, 10^−1^ to 10^−6^ for the other agents) were applied in six replicates in the 96-well plate with the pre-formed stationary culture of Vero E6 cells (density 20,000 cells/well), produced 24 h prior to infection. After the adsorption step (1 h at 37 °C, 5% CO_2_), the inoculum was removed, and medium 199 (supplemented with 2% FBS and 2 mM of L-glutamine) was added. After incubation (72 h at 37 °C, 5% CO_2_), the presence of CPE was recorded, and TCID_50_/mL was calculated. The negative control cutoff for temperature samples was 0.5 log TCID_50_/mL. However, the cutoff for the other agents was 2.5 log TCID_50_/mL for GLU, 1.7 and 1.5 log TCID_50_/mL for 0.5 and 1.0 mg/mL ASC, and 1.8 log TCID_50_/mL for βPL.

### 2.5. Virus Quantification by Plaque Assay

In order to verify the absence of plaque in the βPL-inactivated samples and quantify the viral stock used in animal challenge, 24-well plates with pre-formed monolayers of Vero CCL-81 cells were inoculated with 4-fold serial dilutions of the samples (10^0^ to 10^−6^, in duplicate). After 1 h of adsorption (37 °C, 5% CO_2_), the inoculum was removed, and 1 mL of semi-solid medium, produced from medium 199 (supplemented with 5% FBS, 40 µg/mL of gentamicin sulfate, and 1 µg/mL of amphotericin B (Gibco, Billings, MA, USA)), and 1.5% medium viscosity carboxymethylcellulose sodium salt (Sigma-Aldrich, Saint Luis, MO, USA) were added to each well, and the plates were incubated for 72 h at 37 °C, 5% CO_2_. Cell monolayers were fixed by adding 1 mL of 1.25% formaldehyde solution (Merck, Darmstadt, HE, Germany) to each well and incubating the plates for at least 90 min (final concentration of 0.625% formaldehyde). Following the fixation step, the plates were washed and stained with 0.4% crystal violet solution (Sigma-Aldrich, Saint Luis, MO, USA). Plaque counts were performed, and the viral titer was calculated in Log_10_ PFU/mL.

### 2.6. Viral Genome Quantification by Real-Time PCR (RT-qPCR)

Nucleic acid purification was performed using the QIAamp Viral RNA Mini Kit (QIAGEN, Hilden, NW, Germany) for oropharyngeal swab or viral culture, and the IndiSpin Pathogen Kit (INDICAL BIOSCIENCE GmbH, Leipzig, SN, Germany) for tissue samples. Briefly, 140 µL of oropharyngeal swab or viral culture supernatant was added to 560 µL of lysis buffer. For tissue samples, fragments of 25–30 mg were disrupted in 1 mL of PBS using TissueRuptorII (QIAGEN, Hilden, NW, Germany), and 200 µL of homogenized sample was added to 20 µL of proteinase K and 100 µL of lysis buffer. All specimens were mixed by vortexing, incubated for 10 min at room temperature, and stored at −80 °C for 24 h. The next RNA extraction steps were carried out according to the manufacturer’s instructions, for each kit. A standard curve was constructed using a commercial plasmid vector 2019-nCoV_N_Positive Control (10006625, IDT, Coraville, IA, EUA) containing a 1260 bp sequence from the SARS-CoV-2 nucleoprotein gene. To quantify samples via RT-qPCR assays, the standard curve was serially diluted ten-fold in 7 Log_10_−2 Log_10_ RNA copies/µL. Monoplex reactions for N1 (Forward: 5′GACCCCAAAATCAGCGAAAT3′, Reverse: 5′TCTGGTTACTGCCAGTTGAATCTG3′, and probe: 5′FAM-ACCCCGC ATTACGTTTGGTGGACC-BHK3′) and N2 (Forward: 5′TTACAAACATTGGCCGCAAA3′, Reverse: 5′GCGCGACA TTCCGAAGAA3′, and probe: 5′FAM-ACAATTTGCCCCCA GCGCTTCAG-BHK3′) target detection were setup with 0.5 µM of each primer, 0.125 µM of TaqMan fluorogenic probe, TaqMan Fast Virus 1-Step Master Mix (Thermo Fisher Scientific, Walthan, MA, USA), and 5 µL of template in a 20 µL final volume, following the CDC protocol [28]. Thermal conditions were 50 °C, 5 min, 95 °C, 20 s, and 40 cycles of 95 °C, 3 s, and 60 °C, 33 s.

### 2.7. Serial Passages (SP)

To evaluate each inactivation protocol and confirm the process efficiency, the selected samples and the respective controls were inoculated using a 20-fold non-cytotoxic dilution (500 µL to 10 mL of final volume in culture medium) in T-25 cell culture flasks with the pre-formed stationary culture of Vero E6 cells (density 100,000 cells/cm^2^), produced 24 h prior to infection, and incubated for 3 or 4 days at 37 °C, 5% CO_2_. After incubation, cell monolayers were examined for CPE and the supernatant was tested for viral RNA using RT-qPCR (see Section 2.6) for viral RNA quantification after each passage. The supernatant of the previous passage (500 µL to 10 mL of final volume) was used to infect a new set of T-25 culture flasks containing pre-formed monolayers. This protocol was repeated for five serial passages [29]. For the control containing non-inactivated virus, it was expected that the virus would continue replicating, and the quantification of the viral genome would not vary over the passages. However, for inactivated samples, it was expected that the virus would not replicate, and the quantification of the viral genome would be reduced with each dilution as the passages proceeded. After a few passages, the residual genome of the inactivated virus became undetectable, proving the viral inactivation.

### 2.8. Ultrastructural Analysis of SARS-CoV-2 Particles by Negative Staining Technique

One drop of the viral suspension was applied onto a formvar-covered electron microscope 400-mesh copper grid (Electron Microscopy Sciences, Hatfield, PA, USA). After 15 s, the excess sample was removed with filter paper, and a drop of 2% phosphotungstic acid (PTA), pH 7.0, was applied. The excess of PTA was removed after 15 s, and the grid was examined by a Hitachi HT 7800 (Hitachi, Chiyoda, Tokyo, Japan) transmission electron microscope (TEM) [30,31]. For biosafety level reasons, 1% glutaraldehyde diluted in sodium cacodylate buffer (1:1) was added to non-inactivated SARS-CoV-2 samples prior to grid preparation and TEM analysis.

### 2.9. Enzyme-Linked Immunosorbent Assay (ELISA)

For all ELISA measurements, the total protein concentration of viral productions was obtained by protein dosage by the BCA method (Pierce BCA Protein Assay Kit, Thermo Scientific, Walthan, MA, USA), being: 744.9 µg/mL for Wuhan produced with FBS, 560.8 µg/mL for Wuhan produced in serum-free medium, 738.7 µg/mL for Gamma (P.1), 729.3 µg/mL for Zeta (P.2), and 710.6 µg/mL for Alpha (B.1.1.7).

To screen for different antibodies, a Maxisorp plate (Nunc, USA Scientific, Ocala, FL, USA) was adsorbed with 100 µL of serum-free virus (SARS-CoV-2 INT) containing 56.08 µg of total protein, and incubated overnight at 4 °C. The wells were then washed once with a wash solution (PBS/0.05% Tween) and then blocked with 5% non-fat dry milk in PBS buffer (1 h at 37 °C). Wells were washed once more before being incubated with two different mouse neutralizing monoclonal antibodies (mAbs), NAb/RBD-1 and NAb/RBD-2, both anti-RBD domain and developed by the Immunological Technology Laboratory of Bio-Manguinhos/FIOCRUZ (hybridoma technology). For the comparison, two commercial mouse anti-Spike-neutralizing mAbs, 40591-MM43 and 40592-MM57 (Sino Biological, Houston, TX, USA), were used. The antibodies were diluted in PBS, ranging from 20 to 0.3125 µg/mL (2-fold serial dilutions, in duplicates), and incubated for 2 h at 37 °C (100 µL final volume). Before incubation with peroxidase-conjugated anti-mouse IgG (Sigma-Aldrich, Saint Luis, MO, USA), the wells were washed 3 times with wash solution. After 1 h of incubation at 37 °C, the wells were again washed 3 times, and TMB substrate (T0440, Sigma-Aldrich, Saint Luis, MO, USA) was added. Afterward, the plate was incubated in the darkness for 30 min. The assay was finished by adding 1 N HCl to stop the reaction that was read at 450 nm. For SARS-CoV-2 batches produced in the presence of FBS, the antibodies Nab/RBD-1 and Nab/RBD-2 were used at a concentration of 20 µg/mL, following the same protocol previously described.

For sandwich ELISA, Maxisorp plates were adsorbed with 5 µg/mL of SARS-CoV-2 Spike-Neutralizing Antibody, Rabbit MAb (40592-R001, Sino Biological, Houston, TX, USA), and incubated overnight 4 °C. The washing and blocking steps followed, as described above. Wells were washed once more before being incubated with different SARS-CoV-2 strains. Then, 200 µL of each virus strain was added in the first well, and 100 µL was transferred to the next well and mixed with 100 µL of PBS. A serial dilution (2-fold, in duplicates) was performed until the last well, and the plates were incubated for 2 h at 37 °C. After incubation, the wells were washed 4 times with wash solution and then incubated with NAb/RBD-1 and NAb/RBD-2. The antibodies (20 µg/mL) were diluted in PBS, and incubated for 1 h at 37 °C. The negative controls (medium with and without serum) were used. Before incubation with peroxidase-conjugated anti-mouse IgG Fc (A0168, Sigma-Aldrich, Saint Luis, MO, USA), the wells were washed 4 times with wash solution, and TMB substrate was added. The assay was finished as described previously, and the results were presented as a function of the total protein concentration in each viral batch. 

### 2.10. Immunochromatographic Test for SARS-CoV-2 Nucleocapsid Protein

CHEMBIO TR DPP^®^ COVID-19 AG Bio-Manguinhos (Bio-Manguinhos, Rio de Janeiro, RJ, Brazil) is a qualitative immunochromatographic test based on the binding of the SARS-CoV-2 nucleocapsid (N) protein to a conjugated antibody (anti-N). To assess the recognition of the N protein, three drops of the run buffer were added to 75 µL of each viral batch. Then, 120 µL of the mixture was added to the sample well. After 10 min, 7 drops of the run buffer were added to the buffer well and the result was revealed within 10 min, as described in the manual of the kit.

### 2.11. Non-Clinical Trials

The non-clinical study protocols were approved by the Institutional Committee of Animal Care and Experimentation (CEUA/FIOCRUZ: LW-17/20) and conducted in strict accordance with the recommendations from the Guide for Care and Use of Laboratory Animals of the Brazilian Society of Science in Laboratory Animals (SBCAL) and the National Council for the Control of Animal Experimentation (CONCEA, Rio de Janeiro, RJ, Brazil). Here, 24 K18-hACE2 knockout mice (males and females), 17- to 20-weeks-old, weighing 18–23 g, SARS-CoV-2-naïve and captivity colony-born at the Institute of Science and Technology in Biomodels of FIOCRUZ (Rio de Janeiro, RJ, Brazil), were used in this study. K18-hACE2 mice (*n* = 12) were intramuscularly (IM) immunized, using a homologous prime-boost dose, 2 weeks apart, with 100 μL of SARS-CoV-2 INT adjuvanted with AddaS03^TM^ (InvivoGen, San Diego, CA, USA) in a volume of 1:1, adjuvant:antigen. Each dose had 280.4 µg of total protein (Pierce BCA Protein Assay Kit, Thermo Fisher Scientific, USA). The remaining K18-hACE2 mice (*n* = 12) were mock-immunized with PBS (negative control group). Animals (*n* = 11) were challenged intranasally (IN) with 10 µL (5 µL in each nostril) of SARS-CoV-2 (1.0 × 10^5^ PFU/dose) two weeks post-immunization and then followed-up for one week, recording the weight and mortality daily. After virus challenge, oropharyngeal swabs (3, 6, and 14 dpi), lungs, and brains (6 and 14 dpi) were collected for SARS-CoV-2 RNA quantification by RT-qPCR (see Section 2.6). Serum samples were collected from immunized animals before and after each dose, as well as after challenge (6 dpi and 14 dpi), and specific neutralizing antibodies (NAb) against SARS-CoV-2 (Wuhan strain) were measured using classical PRNT_50_ (see Section 2.12). Euthanasia was performed at the following time points: pre-challenge (*n* = 1/group), 6 dpi (*n* = 10/mock-immunized group; *n* = 8/immunized group), and 14 dpi (*n* = 3/immunized group). Histopathology was used to evaluate the extent of tissue damage in the lungs and brains of mice. Statistics were assessed via one-way ANOVA using the Kruskal–Wallis test and Dunn’s post-test, on GraphPad Prism 5 (GraphPad Software, San Diego, CA, USA), to compare the oropharyngeal swab, lung, and brain sample results. Body weight changes between mock-immunized and SARS-CoV-2 INT-immunized mice 14 days post-infection were assessed by the two-way ANOVA test using the Bonferroni post-test, on GraphPad Prism 5.

### 2.12. Plaque Reduction Neutralization Test (PRNT)

Individual serum samples from all groups were pooled prior to challenge for NAb quantification, and pre-immunization samples were used for baseline analyses. Samples collected post-challenge were individually analyzed. Previously inactivated (56 °C, 30 min) serum samples were serially diluted in culture medium from 1:6 to 1:1458 (3-fold dilution factor), and PRNT_50_ was performed in Vero CCL-81 cells (density 200,000 cells/well) cultivated in 24-well plates using the Wuhan strain. The results were expressed in reciprocal serum dilution [32]. PRNT_50_ titers less than 1:10 were considered negative for NAb presence, and the upper limit of quantification of positive samples was 1:1458. 

## 3. Results

### 3.1. Selecting the Most Effective Inactivation Protocol

Here, we subjected SARS-CoV-2 (Wuhan strain) to different heat and chemical treatments. The parameters shown in Table 1 were followed for each inactivating agent to select the more efficient protocol to inactivate SARS-CoV-2 that met the main predetermined selection criteria (see Section 2.3). Initially, we produced a SARS-CoV-2 stock in Vero E6 cell culture with FBS. In this condition, SARS-CoV-2 replicated optimally, yielding 10.81 and 10.94 Log_10_ RNA copies/mL for N1 and N2, respectively, and 7.37 Log_10_ PFU/mL (5.89 Log_10_ TCID_50_/mL).

**Figure 1:**
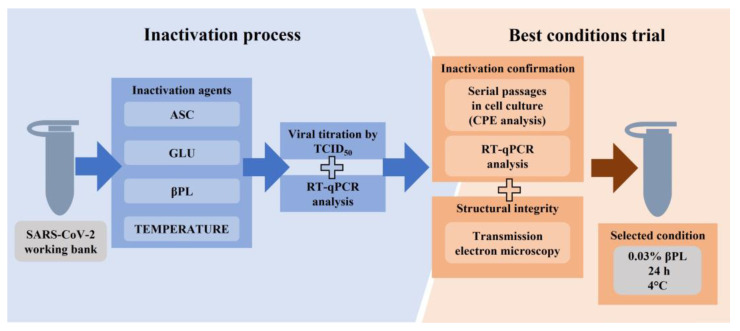
Workflow for selecting an inactivation agent. SARS-CoV-2 (Wuhan strain) inactivation was performed using a working bank produced with fetal bovine serum. The efficacy was preliminarily assessed for all five agents using the TCID_50_ assay and RT-qPCR. For inactivation confirmation, the promising conditions were subjected to five serial passages in cell culture combined with the cytopathic effect (CPE) and RT-qPCR analysis. The most effective condition was selected to inactivate the other working banks. ASC, ascorbic acid; GLU, glutaraldehyde; βPL, beta-propiolactone.

After the first round of inactivation tests, the residual infectivity of samples and viral RNA quantification were analyzed by TCID_50_ and RT-qPCR, respectively. Promising samples were selected for the next step, being subjected to five serial passages in Vero E6 cells. CPE was then evaluated at each serial passage, as well as the detection of viral RNA from the culture supernatants. Finally, the integrity of the viral particles was analyzed by TEM. The workflow for selecting the best experimental condition is described in Figure 1.

**Figure 2:**
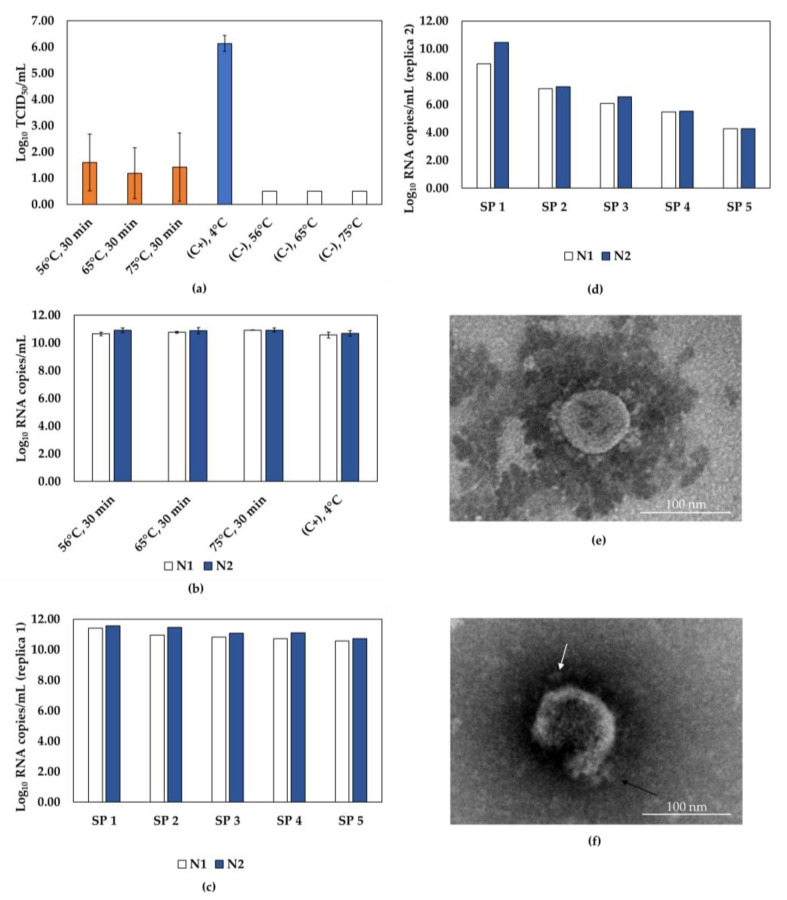
Efficiency of SARS-CoV-2 inactivation by heat. (**a**) SARS-CoV-2 infectivity incubated at different temperatures (56 °C, 65 °C, and 75 °C) for 30 min, as assessed by TCID_50_ assays. Inactivated samples represented by orange bars, non-inactivated virus control (C+) represented by the blue bar, and negative control (C-, inactivating agent) represented by white bars. (**b**) RT-qPCR quantification of molecular targets N1 (white bars) and N2 (blue bars) in heat-inactivated samples, and (**c**,**d**) samples from 5 serial passages of SARS-CoV-2 incubated at 65 °C, 30 min (independent replicas 1 and 2). (**e**) Representative transmission electron microscopy (TEM) image of SARS-CoV-2 untreated, and (**f**) SARS-CoV-2 subjected to 65 °C, 30 min. Spikes of the viral particle are indicated by arrows. Scale bar, 100 nm; SP, serial passage; C+, positive control; C-, negative control.

We submitted SARS-CoV-2 samples for inactivation at 56 °C, 65 °C, or 75 °C for 30 min, but conflicting results were observed (Figure 2). Although TCID_50_ data indicated inactivation over 99.99% and a reduction of at least 3.94 Log_10_ TCID_50_/mL under all conditions tested (Figure 2a), serial passage results showed that inactivation of 5 mL at 65 °C for 30 min was effective in one replica, but ineffective in the next (Figure 2c,d). In addition, despite slight variations in the RNA quantification (Figure 2b), partially destroyed particles were observed in the condition that achieved complete inactivation (Figure 2f), in contrast to the integrity shown for non-inactivated SARS-CoV-2 (Figure 2e). The volume used (5 mL) could have been a problem for heat transfer, thus compromising the reproducibility of the tests. To address this issue, the same heat inactivation protocols were used with a smaller volume of viral suspension (0.3 mL); however, this strategy showed similar conflicting results, indicating that heat was not the best protocol for inactivating SARS-CoV-2 samples.

Combined with Cu(SO_4_)_2_, which promotes its oxidation, ASC was used as a possible SARS-CoV-2-inactivating agent. Under the conditions tested, the quantification of viral RNA was not compromised; however, ASC combined with Cu(SO_4_)_2_ was ineffective in completely inactivating SARS-CoV-2. Even at the highest concentration and for the longest period time (1 mg/mL, 96 h), residual infectious SARS-CoV-2 was still detected (29.6%), showing a reduction of only 0.53 Log_10_ TCID_50_/mL. As a result, ASC was discarded as an inactivating agent and did not proceed to the next step.

**Figure 3:**
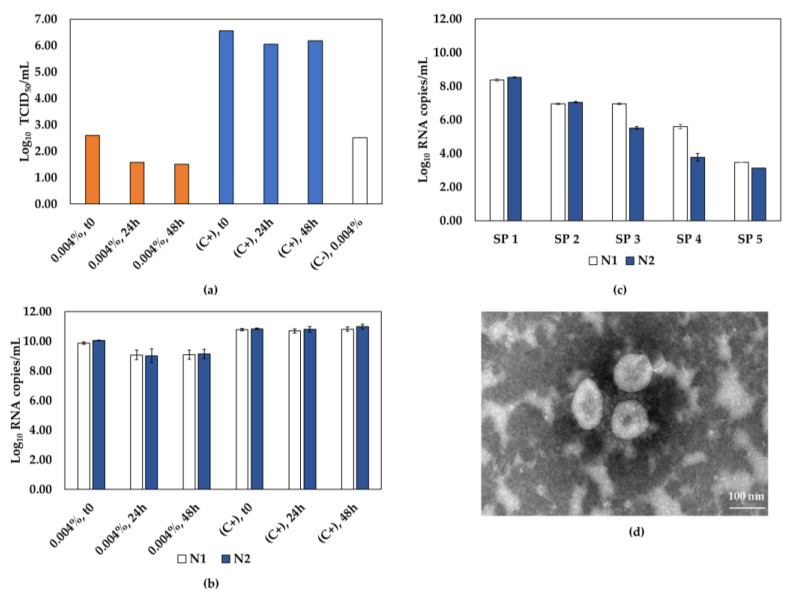
The effectiveness of glutaraldehyde in inactivating SARS-CoV-2. (**a**) SARS-CoV-2 infectivity of samples incubated with 0.004% glutaraldehyde, for 0, 24, and 48 h, as determined by TCID_50_ assays. Inactivated samples represented by orange bars, non-inactivated virus control (C+) represented by blue bars, and negative control (C-, inactivating agent) represented by the white bar. (**b**) RT-qPCR quantification of molecular targets N1 (white bars) and N2 (blue bars) in glutaraldehyde-inactivated samples and (**c**) samples from 5 serial passages of SARS-CoV-2 incubated with 0.004% glutaraldehyde for 24 h. (**d**) Representative TEM image of SARS-CoV-2 incubated with 0.004% glutaraldehyde for 24 h. Scale bar, 100 nm; SP, serial passage, C+, positive control; C-, negative control.

We also used longer incubation times to inactivate SARS-CoV-2 by GLU and analyzed the decrease of viral titer as well as particle morphology. As presented in Figure 3a, a reduction of at least 3.48 Log_10_ TCID_50_/mL was achieved in all conditions tested, indicating an inactivation above 99.97%. However, a decrease of up to 1.8 Log_10_ RNA copies/mL was also observed when compared to the controls (Figure 3b). Serial passages were performed on samples from the best condition for this agent: 0.004% GLU for 24 h. No CPE was observed, and viral RNA detection decreased at each passage, confirming the absence of viral replication, as shown in Figure 3c. A representative TEM image of the inactivated virus is shown in Figure 3d, evidencing that GLU inactivation preserved the viral particle integrity compared to non-inactivated SARS-CoV-2 (Figure 2e). However, due to the reduced detection of viral RNA, it did not prove to be the best inactivation method for SARS-CoV-2.

**Figure 4:**
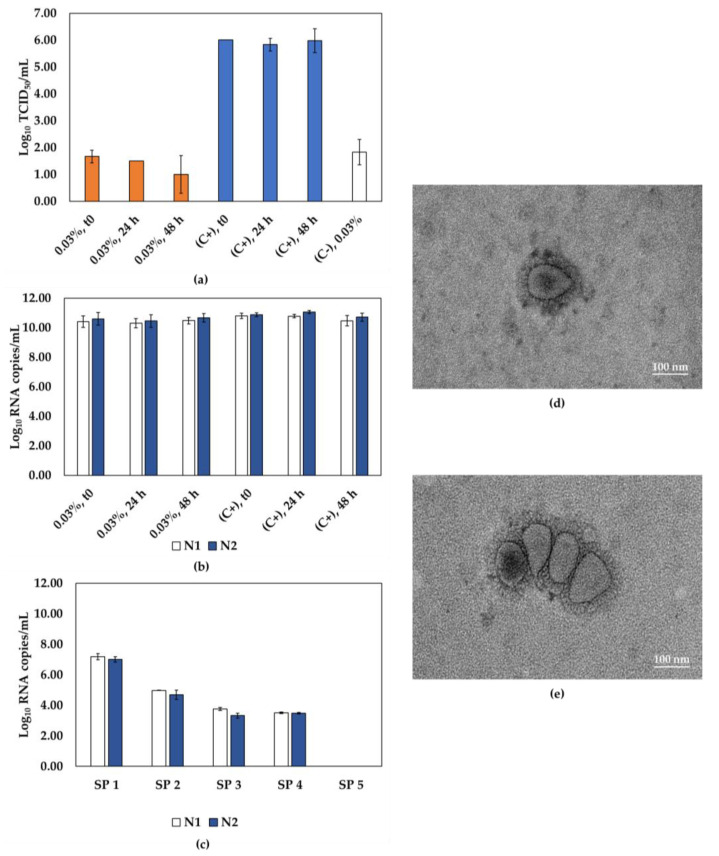
The effectiveness of βPL in inactivating SARS-CoV-2. (**a**) SARS-CoV-2 infectivity of samples incubated with 0.03% βPL for 0, 24, or 48 h, as determined by TCID_50_ assays. Inactivated samples represented by orange bars, non-inactivated virus control (C+) represented by blue bars, and negative control (C-, inactivating agent) represented by the white bar. (**b**) RT-qPCR quantification of molecular targets N1 (white bars) and N2 (blue bars) in samples subjected to inactivation by βPL, and (**c**) samples from 5 serial passages for SARS-CoV-2 incubated with 0.03% βPL for 24 h. (**d**,**e**) Representative TEM images of SARS-CoV-2 incubated with 0.03% βPL for 24 h. Scale bar, 100 nm; SP, serial passage, C+, positive control; C-, negative control.

Finally, the samples were treated with βPL, and the results showed high levels of SARS-CoV-2 inactivation (Figure 4). Besides that, for the three conditions evaluated, viral RNA levels were comparable to the controls (Figure 4b). The samples submitted to 0.03% βPL for 24 h showed a reduction of 4.36 Log_10_ TCID_50_/mL, indicating an inactivation above 99.99%. The residual infectivity of the βPL-inactivated samples was also assessed by the plaque assay, and indeed, no cytopathic effect was observed, indicating complete inactivation (Figure S1). Here, we also observed a cytotoxic effect of the undiluted inactivated samples, but a 4-fold dilution was sufficient to minimize the cytotoxicity impacts of βPL, as visualized in the cell monolayers of the plaque assay (Figure S1). The samples subjected to this condition proceeded to the next step and showed no CPE through serial passages. Moreover, RNA detection decreased over the course of five serial passages, indicating that the virus was effectively inactivated (Figure 4c). Representative TEM images of the inactivated virus indicate that, despite the presence of aggregates, our βPL inactivation methodology preserved the viral particle integrity (Figure 4d,e) compared to non-inactivated SARS-CoV-2 (Figure 2e).

Together, these findings indicated that 0.03% βPL for 24 h is the most effective inactivation protocol, and it was then used to successfully inactivate SARS-CoV-2 variants of epidemiological relevance, such as Alpha (B.1.1.7 lineage), Gamma (P.1 lineage), and Zeta (P.2 lineage). Once again, there was a reduction of at least 4.17 Log_10_ TCID_50_/mL compared to the positive control of each variant, showing an inactivation of over 99.99% (Figure S2). The absence of plaques and a decrease in the quantification of viral RNA after five serial passages corroborated this result, indicating complete inactivation of the SARS-CoV-2 variants (Figures S1 and S2).

### 3.2. Functional Characterization of Inactivated SARS-CoV-2

Based on the results of the first round of inactivation tests and subsequent serial passages, the methodology of chemical inactivation by βPL (0.03%, 24 h) was also adopted for a serum-free SARS-CoV-2 batch (Wuhan strain, SARS-CoV-2 INT), whose yield was 6.71 Log_10_ PFU/mL (10.09 and 10.18 Log_10_ RNA copies/mL for N1 and N2, respectively). Titration assays and serial passage analysis confirmed the inactivation of the sample, allowing it to be safely used in functional characterization assays in BSL-2 facilities.

**Figure 5:**
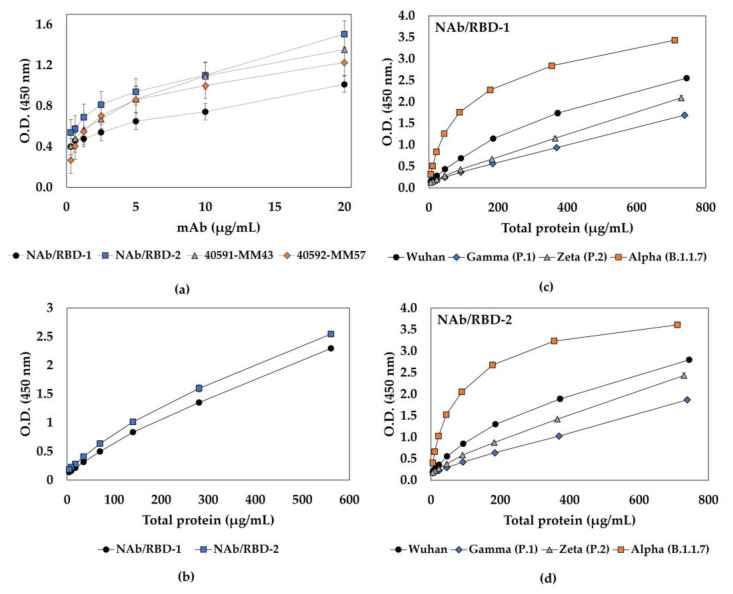
SARS-CoV-2 interaction with mouse neutralizing monoclonal antibodies (mAbs). (**a**) Functional characterization of inactivated SARS-CoV-2 INT (serum-free), assessed by ELISA, using four different mouse neutralizing mAbs: NAb/RBD-1 (●) and NAb/RBD-2 (■), both anti-RBD, and 40591-MM43 (▲) and 40592-MM57 (◆), both anti-Spike. (**b**) Sandwich ELISA of SARS-CoV-2 INT using NAb/RBD-1 (●) and NAb/RBD-2 (■). Sandwich ELISA of virus batches produced in the presence of SFB, Wuhan (●), Gamma (◆), Zeta (▲), and Alpha (■), using (**c**) NAb/RBD-1 and (**d**) NAb/RBD-2 for virus detection.

To assess the functionality of the βPL-inactivated material and screen for the most efficient antibodies, ELISA was used to evaluate the interaction of SARS-CoV-2 INT with different commercial or in-house neutralizing mAbs for Spike protein. It is possible to note that all mAbs could recognize the inactivated virus (Figure 5a). However, antigenicity analysis of the SARS-CoV-2 batches produced in the presence of SFB (Wuhan strain and variants Alpha, Gamma, and Zeta) showed reduced absorbance (Figure S4) when compared to the SARS-CoV-2 INT batch. To increase the sensitivity of the test, a sandwich ELISA was performed to evaluate the interaction of five batches of inactivated SARS-CoV-2 (produced in the presence or absence of serum), and the results showed that two different selected mAbs were able to bind to the inactivated viruses in a dose-dependent manner (Figure 5b–d). This indicates that the βPL inactivation methodology proposed in this study did not compromise antigen–antibody binding since the antigenicity of the inactivated particles was maintained.

Whereas inactivation by βPL may also affect the nucleocapsid protein (N), a qualitative antigen test was used to evaluate the recognition of the N protein of SARS-CoV-2 (Wuhan strain) and the variants Alpha (B.1.1.7), Gamma (P.1), and Zeta (P.2). The data showed that βPL inactivation did not prevent N protein recognition of all SARS-CoV-2 strains analyzed (Figure S3).

### 3.3. Immunogenicity of Inactivated SARS-CoV-2

**Figure 6:**
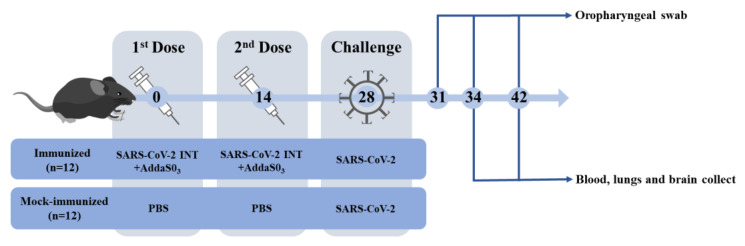
Non-clinical trial timeline. K18-hACE2 mice were divided into two groups and immunized with 2 doses, 14 days apart, and challenged 14 days after the second dose. Serum samples were collected before and after each dose, as well as 34 (6 dpi) and 42 (14 dpi) days after challenge for the analysis of specific NAbs against SARS-CoV-2 by classical PRNT_50_. After challenge, oropharyngeal swabs (3, 6, and 14 dpi), lungs, and brains (6 and 14 dpi) were collected to assess SARS-CoV-2 RNA levels using RT-qPCR. SARS-CoV-2 INT, βPL-inactivated serum-free SARS-CoV-2.

We directly explored the protective efficacy of K18-hACE2 mice elicited by SARS-CoV-2 INT two-dose immunization to use it as a positive control in non-clinical trials, as illustrated in Figure 6.

**Figure 7:**
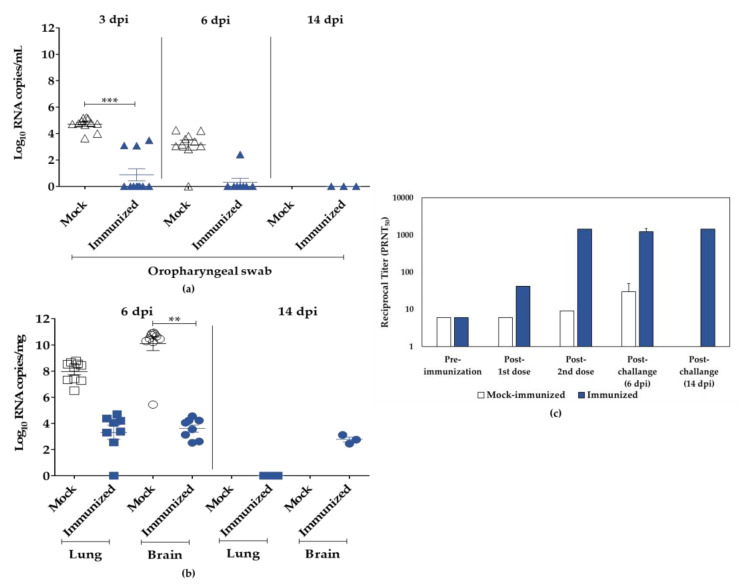
Viral RNA and neutralizing antibodies’ (NAbs) quantification of samples from K18-hACE2 mice. (**a**) Viral titers in oropharyngeal swabs were estimated by viral RNA copy numbers per mL, collected from mice at 3, 6, and 14 dpi. (**b**) Viral titers in lungs and brains of infected mice were estimated as genomic RNA copy number per mg at 6 or 14 dpi. (**c**) Serum samples were collected from immunized animals before and after each dose and after challenge (6 dpi and 14 dpi) for PRNT_50_ analysis, and NAb titers were expressed as reciprocal serum dilution. Mock group represented by white symbols and bars, and immunized group represented by blue symbols and bars. Asterisks (*) represent statistical significance by the ANOVA test, where ** *p* ≤ 0.01, and *** *p* ≤ 0.001; if (ns, not shown), *p* > 0.05.

When compared to the mock, immunized animals had a 100% survival rate and no clinical signs until euthanasia at 6 (*n* = 8) and 14 dpi (*n* = 3). In the control group, one animal died at 5 dpi, and most animals (*n* = 6) showed clinical signs, such as arched back, respiratory distress, and paralysis at 6 dpi, when all animals were euthanized. From the 4th to 6th dpi, body weight changes were statistically different between immunized (body weight change 0.09% ± 0.88%) and mock-immunized groups (body weight change −4.35% ± 1.58%), as observed in Figure S5. Moreover, the immunized group showed no histopathological lesions in the brain, while all animals in the control group presented encephalitis. Conversely, at the respective endpoint, the histopathological lesions in the lungs were similar in both groups, with bronchopneumonia being the most common symptom. When compared to the respective controls, the immunized group after challenge had lower amounts of SARS-CoV-2 genomic RNA in the oropharyngeal swab, 0 to 3.49 Log_10_ RNA copies/mL (3 dpi) and 0 to 2.41 Log_10_ RNA copies/mL (6 dpi), lungs, 0 to 4.69 Log_10_ RNA copies/mg (6 dpi), and brains, 2.45 to 4.55 Log_10_ RNA copies/mg (6 dpi) (Figure 7a,b).

A virus-neutralization assay was used to assess serum samples from immunized or mock-immunized animals with SARS-CoV-2 INT as well as after challenge (Figure 7c). SARS-CoV-2 INT was able to induce low NAb titers (1:42) detected two weeks after the first dose, followed by a remarkable increase in the levels of NAb (>1:1458) measured after the second dose and immediately before challenge. Furthermore, SARS-CoV-2 challenge induced low NAb titers (1:30) in surviving mock-immunized mice at 6 dpi. In contrast, after challenge, all animals immunized with SARS-CoV-2 INT maintained high NAb levels, with the majority (64%) reaching the maximum PRNT_50_ titer used in this assay.

## 4. Discussion

SARS-CoV-2 cell culture studies must still be conducted in high-containment laboratories [2,3]. However, these laboratories are limited and, especially during the COVID-19 pandemic, they are in huge demand and crowded, with high maintenance and personal protective equipment costs. To minimize this issue, many studies can be safely conducted at lower containment levels if the viral samples are effectively inactivated [2] to protect the healthcare professional from contamination while handling the material.

Here, we tested different methods commonly used to inactivate viruses for use as input for different purposes, such as internal control of analytical assays and non-clinical trials, particularly when the whole viral particle must be preserved. In addition, we also adopted a robust inactivation confirmation protocol combining cell culture and molecular biology techniques.

Particularly for use in molecular assays or as process control, heat-inactivated SARS-CoV-2 (65 °C, 30 min) has been commercially available since the beginning of the pandemic [33,34,35,36,37,38]. Our study attempted heat inactivation in samples from cell culture supernatants using temperatures and times equivalent to or higher than recommended (56 °C, 65 °C, and 75 °C for 30 min). However, the results were inconsistent and did not achieve the desired success after analyzing serial passages. Indeed, different authors have found conflicting results for SARS-CoV-2-inactivation efficiency using similar temperature and time ranges [11,39,40]. The variations between studies may also be related to the methods used to confirm viral inactivation, since different techniques may have different sensitivities and, consequently, provide false-negative inactivation data. In addition, the efficacy of inactivation can be influenced by other factors, including the type and physical state of the sample, matrix composition, heat source, and tube type [12,40]. As a result, the heat inactivation protocols presented in this work are not robust and must be improved.

In comparison to ASC (70.4%), treatment with βPL and GLU at a specific concentration and time resulted in efficient inactivation (>99.97%), even with high virus titers. RT-qPCR detection of viral RNA revealed that βPL was more efficient in maintaining RNA quantification, with a variation < 0.5 Log_10_ RNA copies/mL compared to GLU (>1 Log_10_ RNA copies/mL). In fact, previous studies have shown that GLU can compromise the detection of nucleic acids due to its crosslinking action [25,41].

Despite the efficiency achieved by most of the chemical agents evaluated, only βPL treatment showed complete inactivation, particle integrity, and maintenance of RNA quantification, making this methodology the best strategy of choice for the study. βPL is known for preserving the structure and antigenicity of the virus [25] and has been employed in the preparation of various inactivated vaccines approved so far for SARS-CoV-2 [22,23,24]. In addition, βPL-inactivated SARS-CoV-2 has also been used as a safe and quality input to establish highly sensitive and specific serological methods [42].

SARS-CoV-2 inactivation by βPL has previously been described using varying concentrations of the agent [11,24,43], but a reduction in immunoreactivity has been reported when compared to inactivation promoted by other agents, such as formaldehyde and UV [44]. Depending on the inactivation strategy adopted, antibody recognition may be reduced or even not detected. Indeed, treatment with high concentrations of βPL or for longer periods of time may induce the post-fusion conformation of protein S, and thus lead to aggregation of the viral particles, resulting in insufficient inactivation or a loss of antigenic potential [44,45,46]. However, purification and concentration strategies, such as ultracentrifugation, cannot be dismissed as an artifact, since the morphology of the virus can be affected under these conditions [47]. Here, the integrity of the unpurified, inactivated viral particle was demonstrated, but the presence of aggregates was observed. Nevertheless, neither the inactivation efficiency of the viral particles nor their antigenicity and immunogenicity were prevented.

We employed a lower concentration and shorter exposure period (1:3000, 24 h) and evaluated the immunoreactivity by ELISA of the βPL-inactivated virus. Even though we did not investigate changes in the tertiary structure of protein S, our results showed that different Spike-neutralizing mAbs for SARS-CoV-2 were able to recognize the βPL-inactivated particles with efficiency. Recognition between serum from convalescent patients and formaldehyde- or UV-inactivated SARS-CoV-2 appears to overcome binding to βPL-inactivated SARS-CoV-2 [44]. This difference seems to be related to the higher concentration of βPL used in the study, as there was about a 4-fold reduction when the highest βPL concentration and the longest exposure time were used (1:2000, 36 h).

In non-clinical trials, inactivated SARS-CoV-2 vaccine candidates have been shown to induce high levels of NAb titers [48,49,50,51,52]. In this work, the βPL-inactivated virus was also employed in a non-clinical trial to assess a functional characterization of the antigen, evaluating its ability to produce a robust immune response and its potential use as a positive control in future non-clinical trials. SARS-CoV-2 INT was well-tolerated and highly immunogenic after homologous challenge in K18-hACE2 transgenic mice, eliciting a strong immune response with high levels of neutralizing antibodies. Studies suggest that formaldehyde may be an efficient alternative to βPL for inactivating SARS-CoV-2 [20]. In Swiss mice, formaldehyde-inactivated SARS-CoV-2 was able to elicit higher titers of neutralizing antibodies compared to βPL-inactivated antigen (1:1856 and 1:706, respectively). In our work, K18-hACE2 mice immunized with SARS-CoV-2 INT showed a humoral response equivalent to that previously described for the formaldehyde-inactivated antigen (>1:1458, limit of detection of the test).

Despite inducing complete protection, low levels of genomic RNA were detected at 6 dpi, particularly in the brain of the animals, and lower levels of lung damage were observed, when compared to the control. The concentration of the viral antigen may justify this detection, since previous studies in HFH4-hACE2 mice demonstrated undetectable levels of viral RNA in the lung using higher doses of inactivated antigen, and decreased levels of RNA using lower doses [50].

Taken together, our findings indicate that the inactivation process of SARS-CoV-2 by βPL was efficient, since the decay of viral RNA detection during serial passages directly revealed the inability of the virus to replicate. Thus, we can assure the success of the protocol adopted in this study. Furthermore, maintenance of the conformation and neutralizing epitopes will allow the safe use of the inactivated SARS-CoV-2 in several applications, including downstream processes, immunoassays, and non-clinical trials.

In this scenario, the proposal to include a SARS-CoV-2 INT control group in non-clinical trials may reduce the use of animals over the course of the study, as it increases the reliability of the results generated, leading to a decrease in the need for repeating the trials. Indeed, non-clinical trials with SARS-CoV-2 INT as an assay control have been conducted, and the results presented here are highly reproducible.

## 5. Conclusions

Issues related to biosafety and biocontainment of SARS-CoV-2 will continue to evolve as the pandemic progresses, and methods for working safely using SARS-CoV-2 will require further evaluation. In addition to reducing the risk of accidental contamination, the development of safe and robust inactivation methodologies will allow studies to be conducted at lower levels of biocontainment. The strategies described in this paper may provide a guide for in-house SARS-CoV-2 inactivation in other laboratories. Combining the viral titration method with consecutive serial passages of the virus in cell culture and RT-qPCR is the most secure way to challenge the sample and detect potentially viable particles that are below the detection limit of the infectivity assay used. Inefficient virus treatment can result in incomplete viral inactivation, which can be disastrous, particularly in high-transmissibility samples with a high viral load. Therefore, the attempt to reproduce an inactivation process established by other groups should be undertaken with caution, since a detailed description of experimental protocols is not always reported in scientific publications. These findings highlighted the warning that an inactivation protocol established by another group should not be adopted without using safe methods to confirm batch-to-batch inactivation.

## Figures and Tables

**Table 1:** Inactivation agents and parameters.

Inactivation Agent	Parameter		
Heat	Temperature	Incubation time	Volume
	56 °C	30 min	5.0 mL 0.3 mL
	65 °C		
	75 °C		
	Final concentration	Incubation time	Temperature
Ascorbic acid	0.5 mg/mL	t_0_, 24, 48, 72, 96 h	4 °C
	1 mg/mL		
Glutaraldehyde	0.004%	t_0_, 24, 48 h	4 °C
β-propiolactone	0.03%	t_0_, 24, 48 h	4 °C

## Data Availability

The data presented in this study are available upon request from the corresponding author.

## Supplementary Materials

The following supporting information can be downloaded at: https://www.mdpi.com/article/10.3390/v15071486/s1, Figure S1: Representative images of the plaque assay to analyze the residual infectivity of βPL-inactivated SARS-CoV-2 strains. Figure S2: Efficiency of SARS-CoV-2 variants’ inactivation by βPL. Figure S3: SARS-CoV-2 strains’ recognition by antigen test for N protein. Figure S4: SARS-CoV-2 strains’ interaction with mouse neutralizing monoclonal antibodies (mAbs). Figure S5: Follow-up of K18-hACE2 mice immunized with SARS-CoV-2 INT.
