# Supplementary material for: Inactivated and Immunogenic SARS-CoV-2 for Safe Use in Immunoassays and as an Immunization Control for Non-Clinical Trials
Source: Viruses. 2023 Jun 30;15(7):1486. doi: 10.3390/v15071486 (PMC10386713; doi:10.3390/v15071486)
Supplement: Supplementary file 1 [file viruses-15-01486-s001.zip › viruses-2426287-supplementary.pdf]

Article

Inactivated and immunogenic SARS-CoV-2 for safe use in immunoassays and as an immunization control for non-clinical trials

Supplementary Material

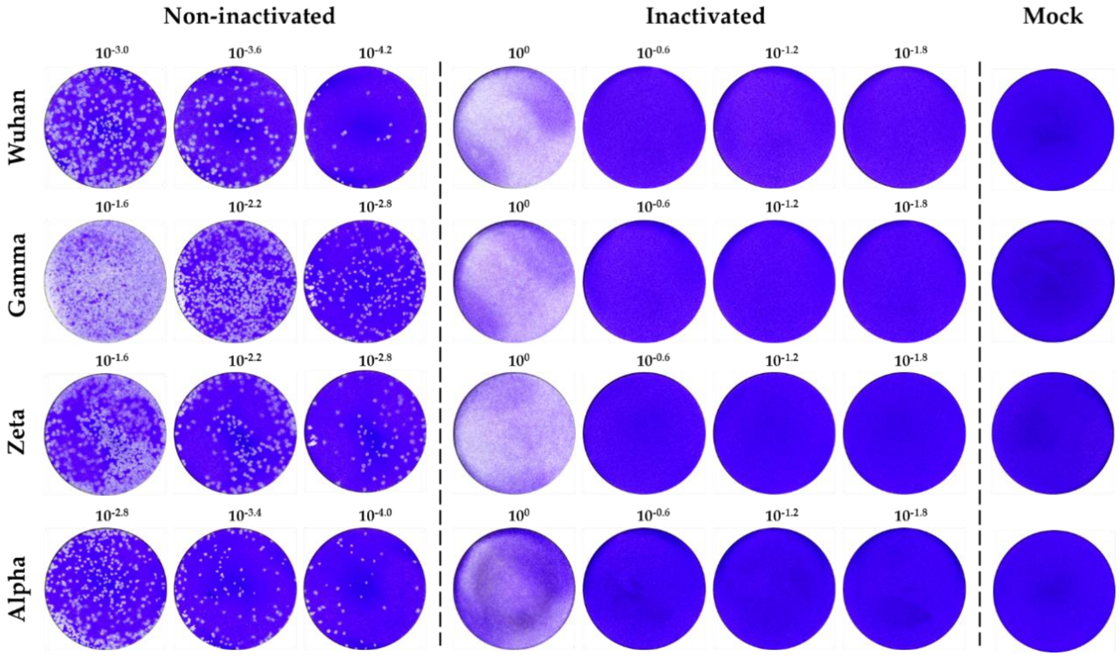

Figure S1. Representative images of the plaque assay to analyze the residual infectivity of βPL-inactivated SARS-CoV-2 strains. Plaque assay in Vero CCL-81 cells of mock (right) and different dilutions of non-inactivated (left) and inactivated (middle) SARS-CoV-2 strains produced in the presence of bovine fetal serum.

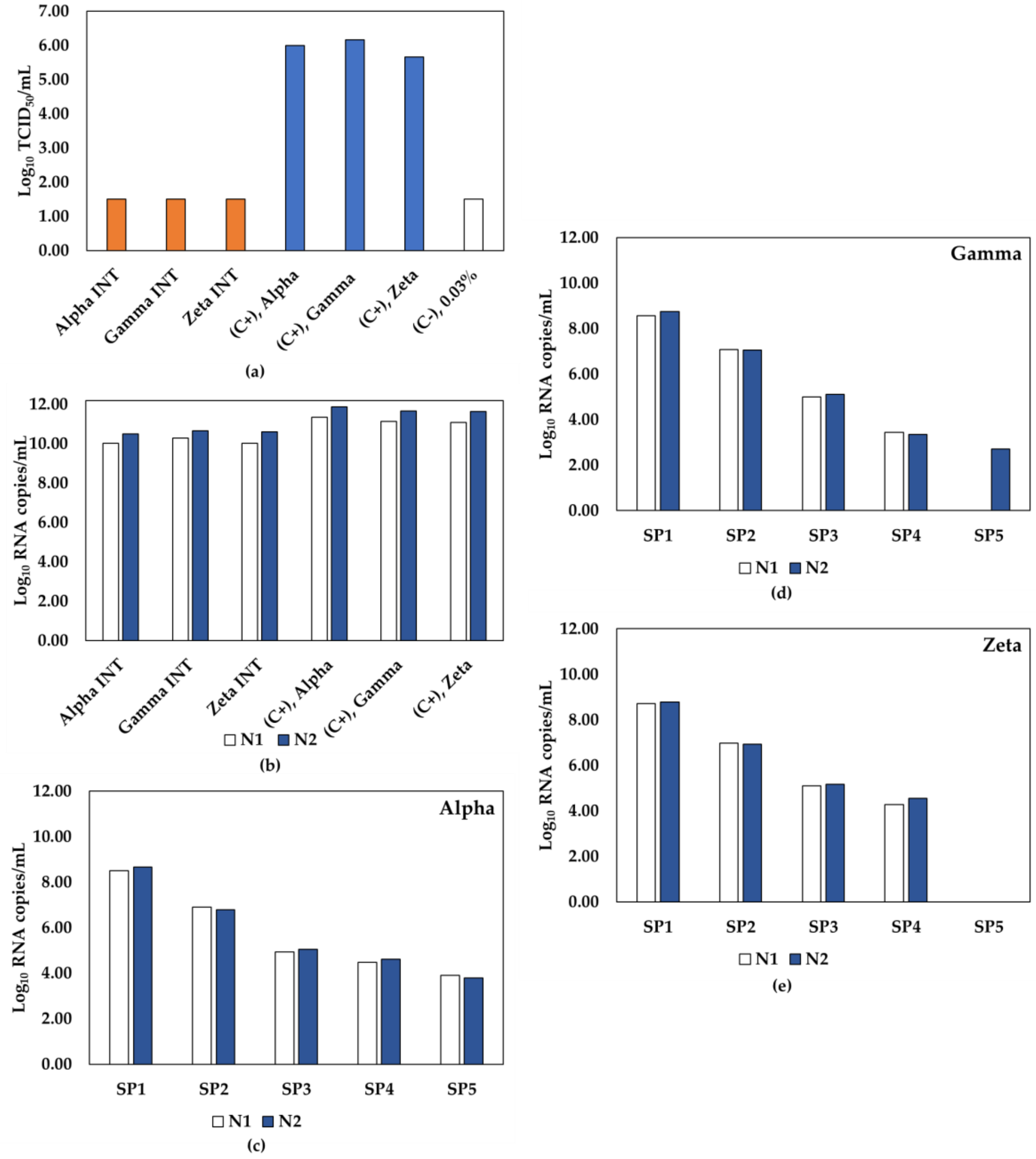

Figure S2. Efficiency of SARS-CoV-2 variants inactivation by βPL. (a) SARS-CoV-2 infectivity of samples incubated with 0.03% βPL for 24 h, as determined by $TCID_{50}$ assays. Inactivated samples represented by orange bars, non-inactivated virus control (C+) represented by blue bars, and negative control (C-, inactivating agent) represented by the white bar. (b) RT-qPCR quantification of molecular targets N1 (white bars) and N2 (blue bars) in samples subjected to inactivation by βPL and (c) samples from 5 serial passages of variants (c) Alpha (B.1.1.7 lineage), (d) Gamma (P.1 lineage), and (e) Zeta (P.2 lineage) incubated with 0.03% βPL for 24 h. SP, serial passage.

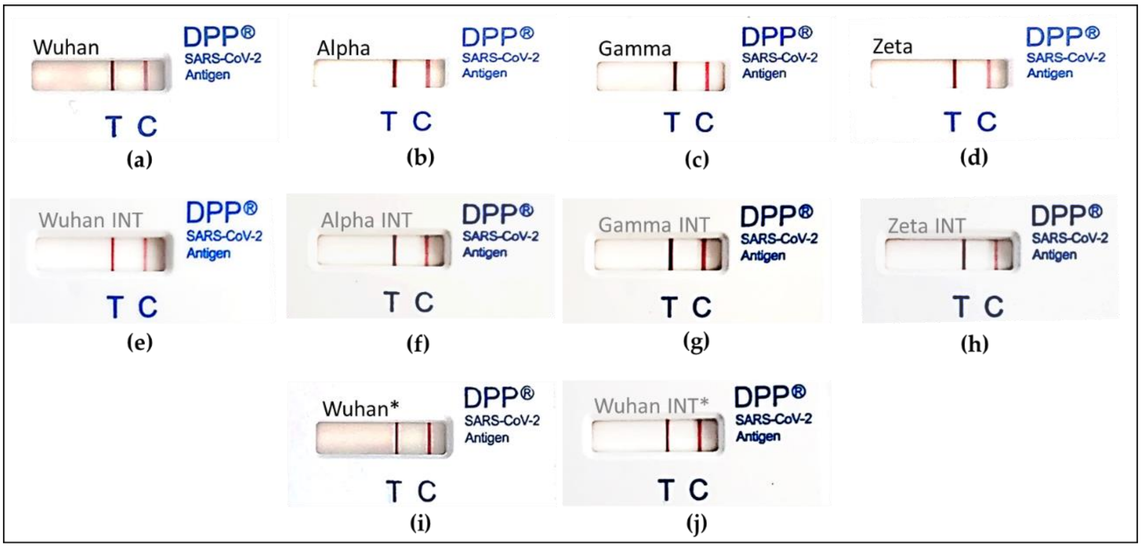

Figure S3. SARS-CoV-2 strains recognition by antigen test for N protein. SARS-CoV-2, Wuhan strain (a, e), and the variants Alpha (b, f), Gamma (c, g) and Zeta (d, h), non-inactivated (black) and inactivated (gray), produced in the absence (*) or presence of 2% fetal bovine serum, were submitted to an antigen recognition test for N protein. C, internal control line indicating that the test was valid; T, sample line indicating recognition of the antigen by the anti-N antibody.

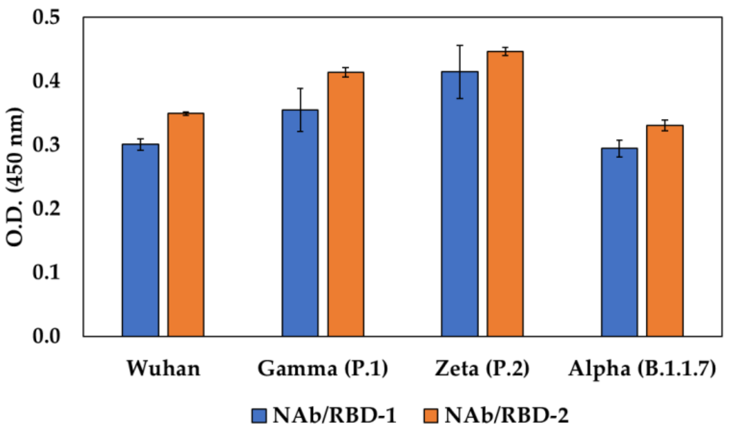

Figure S4. SARS-CoV-2 strains interaction with mouse neutralizing monoclonal antibodies (mAbs). βPL-inactivated SARS-CoV-2, produced in the presence of fetal bovine serum, were submitted to functional characterization by ELISA, using two different mouse neutralizing mAbs: NAb/RBD-1 (blue bars) and NAb/RBD-2 (orange bars), both anti-RBD.

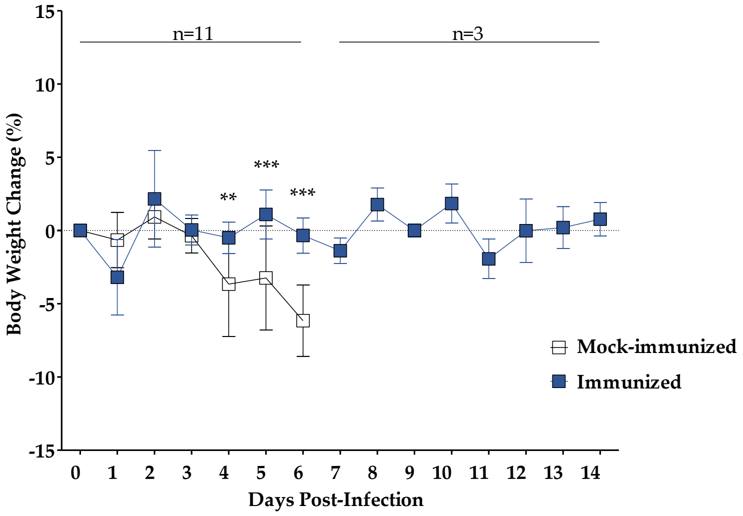

Figure S5. Follow up of K18-hACE2 mice immunized with SARS-CoV-2 INT. Body weight changes between mock-immunized and SARS-CoV-2 INT immunized mice following 14 dpi. Asterisks (*) represent statistical significance by ANOVA test where *, $p \leq 0.05$; **$p \leq 0.01$; ***, $p \leq 0.001$; if (ns), $p > 0.05$ not shown.
